# Exosome-Derived lncRNA NEAT1 Exacerbates Sepsis-Associated Encephalopathy by Promoting Ferroptosis Through Regulating miR-9-5p/*TFRC* and *GOT1* Axis

**DOI:** 10.1007/s12035-022-02738-1

**Published:** 2022-01-17

**Authors:** Xue-biao Wei, Wen-qiang Jiang, Ju-hao Zeng, Lin-qiang Huang, Hong-guang Ding, Yuan-wen Jing, Yong-li Han, Yi-chen Li, Sheng-long Chen

**Affiliations:** 1 Department of Geriatric Intensive Care Unit, Guangdong Provincial Geriatrics Institute, Guangdong Provincial People’s Hospital, Guangdong Academy of Medical Sciences, Guangzhou, People’s Republic of China; 2 Department of Critical Care Medicine, Guangdong Provincial People’s Hospital, Guangdong Academy of Medical Sciences, 106 Zhongshan Er Road, Guangzhou, 510080 People’s Republic of China

**Keywords:** Exosome, NEAT1, Ferroptosis, Sepsis, Encephalopathy

## Abstract

Sepsis can cause sepsis-associated encephalopathy (SAE), but whether SAE was induced or exacerbated by ferroptosis remains unknown. In this study, the rat sepsis model was constructed using the cecal ligation and puncture method. The blood–brain barrier (BBB) permeability was measured by Evans blue dye (EBD) in vivo. The levels of ROS, Fe ion, MDA, GSH, and GPX4 were assessed by enzyme-linked immunosorbent assay (ELISA). The exosomes isolated from serum were cultured with bEnd.3 cells for the in vitro analysis. Moreover, bEnd.3 cells cultured with 100 μM FeCl3 (iron-rich) were to simulate ferroptosis stress. The cell viability was evaluated by Cell Counting Kit-8 (CCK-8) assay. A dual-luciferase reporter gene assay was performed to confirm the relationship between miR-9-5p with NEAT1, *TFRC*, and *GOT1*. In vivo, it is found that BBB permeability was damaged in model rats. Level of ROS, Fe ion, and MDA was increased, and level of GSH and GPX4 was decreased, which means ferroptosis was induced by sepsis. Exosome-packaged NEAT1 in serum was significantly upregulated in model rats. In vitro, it is found that NEAT1 functions as a ceRNA for miR-9-5p to facilitate *TFRC* and *GOT1* expression. Overexpression of NEAT1 enhanced ferroptosis stress in bEnd.3 cells. Increased miR-9-5p alleviated sepsis-induced ferroptosis by suppressing the expression of *TFRC* and *GOT1* both in vivo and in vitro. In conclusion, these findings suggest that sepsis induced high expression of serous exosome-derived NEAT1, and it might exacerbate SAE by promoting ferroptosis through regulating miR-9-5p/*TFRC* and *GOT1* axis.

**Supplementary Information:**

The online version contains supplementary material available at 10.1007/s12035-022-02738-1.

## Introduction

Sepsis is a common life-threatening complication caused by the host’s immune response to infection, including burns, shock, severe infection, severe trauma, and surgery. The severity can be divided into sepsis, severe sepsis, and septic shock [1, 2]. As estimated, the annual diagnosed globally sepsis patients reach 31.5 million, with 17% and 26% be hospitalized as sepsis and severe sepsis, respectively [3]. The predisposing factors of sepsis mainly include age (young or old), pre-existing diabetes, liver cirrhosis, malignant tumors, burns, organ transplantation, long-term use of immunosuppressants, radiotherapy, or long-term indwelling catheters. In recent years, ferroptosis was also reported to be involved in the pathological process of sepsis [4, 5].

Ferroptosis is one type of programmed cell death that is iron-dependent and different from cell necrosis, apoptosis, and autophagy [6, 7]. The essence of ferroptosis is a kind of disorder of lipid oxidation and metabolism under the catalysis of iron ions, which causes weakened antioxidant capacity, accumulated lipid reactive oxygen species (ROS), imbalanced intracellular redox, decreased glutathione (GSH), and glutathione peroxidase 4 (GPX4), thus inducing cell death. Ferroptosis related to the occurrence and development of many diseases, including brain damage [8]. The fatality rate of patients with sepsis is positively correlated with the severity of sepsis-associated encephalopathy (SAE), which is still a clinical problem that needs to be solved urgently [9–11]. Yao et al. [12] demonstrated that hippocampal neuronal ferroptosis involved cognitive dysfunction in rats with SAE through the Nrf2/GPX4 signaling pathway. However, whether neurons ferroptosis is the main reason for SAE remains unknown.

Nuclear-enriched transcript 1 (NEAT1) is a long non-coding RNA that plays a vital role in the cell cycle, proliferation, and apoptosis of tumor cells, with a cancer-promoting effect [13, 14]. Moreover, it was involved in regulating ferroptosis sensitivity in non-small cell lung cancer [13]. One study had found that the expression of lncRNA NEAT1 in the blood of patients with sepsis is significantly increased [15]. However, the role of NEAT1 in sepsis-induced ferroptosis is still unclear. Bioinformatics analysis revealed that NEAT1 binds to hsa-miR-9-5p [16], which targets genes of transferrin receptor (*TFRC*) and glutamic-oxaloacetic transaminase 1 (*GOT1*) [17, 18]. The iron transporter receptor *TFRC* can transport iron ions from outside cell [19]. The iron transporter *GOT1* is the only known iron export protein necessary for iron transport between different types of cells [20]. *TFRC* participates in sulfasalazine-induced ferroptosis in breast cancer cells [21]. As reported, miR-9 inhibits ferroptosis by downregulating *GOT1* expression, resulting in ROS accumulation [22]. *TFRC* and *GOT1* play essential roles in the occurrence of ferroptosis [23, 24]. Therefore, we hypothesized that lncRNA NEAT1 might function as a ceRNA for miR-9-5p to facilitate the expression of *TFRC* and *GOT1*, which accelerate the iron into the brain microvascular endothelial cells, induced ferroptosis, and SAE.

Exosomes are small membrane vesicles (30–150 nm) containing complex molecules, including protein, lipids, coding, or non-coding RNAs. It provides stable environment for therapeutic agent, which transports vesicles and allows therapy to enter the cell directly [25]. Several exosomal lncRNAs (e.g., MALAT1 and Hotairm1) passed through the blood–brain barrier (BBB) and played essential roles in sepsis [26–28]. Therefore, we assumed that lncRNA NEAT1 in the blood might also enter brain microvascular endothelial cells through exosomes.

To test the above hypothesis, the in vivo and in vitro sepsis-induced ferroptosis models were established. The present study aimed to figure out whether exosomal lncRNA NEAT1 affects sepsis-induced ferroptosis through NEAT1/miR-9-5p/*TFRC* and *GOT1* axis. Besides, what is the role of ferroptosis in SAE?

## Material and Methods

### Experimental Animals

Thirty specific-pathogen-free (SPF) male C57BL/6 rats at 8 weeks weighing 200–250 g were obtained from the Experimental Animal Center of Sun Yat-sen University (Guangzhou, China). The rats were housed under room temperatures (25 ± 2 °C and 12-h light/dark cycle) and given water and food ad libitum before the trial. Then, they were randomly assigned into the control group (*n* = 10), model group (*n* = 10), and model + miR-9-5p angomir group (*n* = 10). All animal procedures were performed under the Care and Use of Laboratory Animals guidelines and approved by the Guangdong Academy of Medical Sciences. According to a previous study, sepsis was induced by the cecal ligation and puncture (CLP) method [29]. Briefly, we anesthetized the rats with 5% chloral hydrate (0.6 ml/100 g body weight) and made a 1.5 cm midline incision on the anterior abdomen to expose the cecum. Then, the cecum was ligated at 30%, punctured twice with a No.4 surgical needle to extrude the fecal content. Finally, 1 ml of normal saline was used for resuscitation. The rats in miR-9-5p angomir group were injected with 100 ng/μl miR-9-5p angomir (RiboBio, Guangzhou, China) into caudal vein (model + miR-9-5p angomir). The rats in the control group experienced the same procedure without ligation and puncture.

### Cell Culture

Rat brain microvascular endothelial cell line bEnd.3 purchased from iCell Bioscience Inc. (Shanghai, China) were cultured at 37 °C with 5% carbon dioxide (CO_2_). To select the optimal concentration of iron stimulation, cells were cultured with graded FeCl_3_, including 0 μM, 100 μM, 200 μM, 300 μM, 400 μM, 500 μM, and 600 μM. Then, the in vitro analysis was performed on bEnd.3 cells treated with 100 μM FeCl_3_ (iron-rich group), exosome isolated from control rats (control), model rats (model), and model + miR-9-5p angomir rats (model + miR-9-5p angomir). Moreover, to further investigate the NEAT1 role in ferroptosis, the bEnd.3 cells were transfected by pcDNA3.0 plasmid (Vector), pcDNA3.0-NEAT1 particular sequence (WT NEAT1), and pcDNA3.0-NEAT1 mutation sequence (MUT NEAT1). The miR-9-5p angomir was bought from GenePharma (Shanghai, China).

### Dual-Luciferase Reporter Gene Assay

The target genes of miR-9-5p, including NEAT1, *TFRC*, and *GOT1*, were predicted with TargetScan (http://www.targetscan.org/vert_72/). Promega Dual-Luciferase® Reporter (DLR®) assay was used to verify whether NEAT1, *TFRC*, and *GOT1* were the direct target genes of miR-9-5p. p-NEAT1-wild-type (WT), p-TFRC-WT, and p-GOT1-WT, and the full-length 3′-UTRs of NEAT1, *TFRC*, and *GOT1* were cloned and amplified using psiCHECK-2 (Promega, Madison). The p-NEAT1-mutant (MUT), p-TFRC-MUT, and p-GOT1-MUT were constructed by site-directed mutagenesis. Fluorescence intensity was observed by a fluorescence detector (Glomax20/20, Promega, Collin, 1989).

### Extraction and Identification of Exosomes in Serum

According to the instruction, the exosomes in the serum of control and model rats were extracted by Qiagen miRNeasy Mini Kit (Qiagen, Valencia, CA, USA). The exosomes were labeled by PKH-26 dye (Red Fluorescent Cell Linker Kit), followed by incubated with bEnd.3 cells for 24 h, and finally examined under the fluorescence microscope (Leica, Wetzlar, Germany).

### Cell Viability Assay

The viabilities of cells treated with graded FeCl_3_ were determined using the Cell Counting Kit-8 (CCK-8; CK04; Dojindo Molecular Technology, Kumamoto, Japan) according to the manufacture’s instruction. The cell viability of iron-rich, control, model, and model + miR-9-5p angomir, Vector, WT NET1, and MUT NEAT1 were tested.

### Measurement of BBB Permeability by Evans Blue Dye (EBD)

EBD obtained from Servicebio (Wuhan, China) was used to assess the cerebral cortex permeability in rats of control, model, and model + miR-9-5p angomir with the method described previously [30]. In brief, we injected 18 mg/kg LPS into intraperitoneal, intravenously injected 2% EBD solution in PBS solution (4 ml/kg; MP Biomedicals; Cat No. 151108). The EBD in PBS was allowed to circulate for 10–30 min and been perfused into the right atrium until it became colorless. Then, we isolated the whole brain, dyed with formamide overnight at 50 °C. After that, the whole brains were dried for 1 h at room temperature and weighed. The concentration of formamide dye was quantified at 611 nm spectrophotometrically.

### Enzyme-Linked Immunosorbent Assay (ELISA) Analysis

In vivo, approximately 0.2–1 g of cerebral cortex in control and model rats were isolated, washed with saline at 4 °C, cut into pieces, and grinded into homogenate. The prepared homogenate was centrifuged at 3,000 × *g* for 10–15 min under low temperature.

In vitro, the concentrations of Fe ion, GSH, ROS, GPX4, and malondialdehyde (MDA) in groups of iron-rich, control, model, and model + miR-9-5p angomir, Vector, WT NEAT1, and MUT NEAT1 were measured. Cells were placed at room temperature for 1 h, centrifuged at 1,000–2,000 × *g* at 4 °C for 10 min before the supernatant was collected. Then, a commercial iron assay kit, GSH assay kit, GPX4 assay kit, and MDA assay kit, purchased from Beyotime Biotechnology (Shanghai, China) were used for the level evaluation of Fe iron, GSH, GPX4, and MDA, respectively. The ROS level was measured by employing the 2′,7′-dichlorofluorescein diacetates (Beyotime Biotechnology, Shanghai, China). The cells were placed at 37 °C for 30 min in the dark, harvested with 0.05% trypsin–EDTA solution, suspended in a fresh medium, and finally photographed and observed by a fluorescence microscope (Leica) [31]. Each experiment was tripled.

### Hematoxylin–Eosin (HE) Staining

The HE staining was performed in vivo on cerebral cortex tissues in control, model, and model + miR-9-5p angomir rats. We isolated the cerebral cortex and fixed it with 4% paraformaldehyde in phosphate buffer with a pH of 7.4. Paraffin-embedded tissues were sectioned, deparaffinized, and hydrated. Moreover, HE staining was also conducted on bEnd.3 cells treated with exosomes isolated from rats in the control, model, and model + miR-9-5p angomir groups. The cells were digested by trypsin and sectioned. PBS washed the sections for three times, fixed with 95% ethanol for 20 min, and finally washed by PBS twice. Finally, all slides were stained with HE and imaged using light microscopy.

### Immunohistochemistry

Immunohistochemistry was performed on rat brain microvascular endothelial cells treated with 10% serum exosome collected from control, model, and model + miR-9-5p angomir rats. The paraffin-embedded section of the cerebral cortex was fixed in 4% paraformaldehyde for 24 h and washed with phosphate-buffered saline (PBS). Subsequently, the sections were immersed in 0.5% triton for 20 min, washed with PBS, incubated with 10% goat serum for 30 min, and incubated overnight at 4 °C with primary antibodies of TFRC (ab214039; Abcam, Cambridge, MA, USA) and GOT1 (ab221939; Abcam). Then, the sections were washed by PBS and incubated with the secondary antibody at 37 °C for 30 min. Again, the sections were washed with PBS and deparaffinized using anti-fluorescence quenching mounting tablets containing 2-(4-Amidinophenyl)-6-indolecarbamidine dihydrochloride (DAPI), kept at 4 °C in dark, and watched on a confocal laser microscope (Leica).

### Western Blot Analysis

Western blot analysis was performed on control and model rats to detect the expression of exosome-specific markers, including CD9 and CD63, with TSG01 as the reference. The protein expression levels of TFRC and GOT1 were evaluated in vivo in groups of control and model, as well as in vitro in groups of control, model, iron-rich, model + miR-9-5p angomir, Vector, WT NEAT1, and MUT NEAT1, employing glyceraldehyde-3-phosphate dehydrogenase (GAPDH) protein as the reference. The concentration of proteins was qualified using Pierce™ BCA Protein Assay Kit (no. 23227; Thermo Scientific, USA) according to the manual operation. The standard sodium dodecyl sulfate–polyacrylamide gel electrophoresis (SDS-PAGE) was conducted. Approximately 20 μg protein was electrophoretically separated in 10% SDS separation gel and concentration gel under 100 V and 120 V, respectively. Then, we transferred the gel on a polyvinylidene fluoride (PVDF) membrane, which was washed with 25 ml Tris-buffered saline (TBS) + tween-20 (TBST, Tween-20: TBS = 1:1,000) for 5 min and finally blocked at 4 °C overnight using 5% skimmed milk powder. Subsequently, the samples was incubated with primary antibodies, including rabbit anti-mouse polyclonal antibodies to TSG01 (1:2000; ab125011), CD9 (1:2000; ab92726), CD63 (1:2000; ab193349), TFRC (1:2000; ab214039), GOT1 (1:1000; ab221939) and GAPDH (1:5,000; ab8245) for 1 h. The samples were then incubated in the secondary antibody, horseradish peroxidase (HRP)-labeled goat anti-rabbit IgG (1:20,000, BA1054, BOSTER Inc.) with oscillation at room temperature for 40 min. The PVDF membrane was then incubated with electrogenerated chemiluminescence (ECL) solution (ECL808-25, Biomiga, San Diego, CA, USA) for 1 min and exposed to X-ray film. Image-Pro Plus 6.0 (Media Cybernetics; Silver Springs, MD, USA) was used for the net density value calculation. Each experiment was tripled.

### Quantitative Real-Time Polymerase Chain Reaction (qRT-PCR)

The expression levels of NEAT1, miR-9-5p, *TFRC*, and *GOT1* were assessed in vitro and in vivo by qRT-PCR*.* According to the manual procedure, the total RNA in the exosome and cells was extracted using TRIzol reagent (Invitrogen, Carlsbad, CA, USA). *U6* and *GAPDH* are used as the inner reference gene for miRNA and mRNAs, respectively. The primers of NEAT1, miR-9-5p, *TFRC*, *GOT1*, *U6*, cel-miR-39-3p, and *GAPDH* were designed and synthesized by Sangon Biotech (Shanghai) Co., Ltd. (Shanghai, China) and are listed in Table S1. Exogenous cel-miR-39-3p was used as a reference gene for detection of exosome-derived NEAT1. To assess the expression levels of mRNAs and lncRNA, 1 μg of total RNA was used as a template on Bestar qPCR RT Kit (DBI Bioscience) in a 20 μl system. Amplification of miR-9-5p was performed using PrimeScript™ RT reagent Kit (Takara) following the manufacturer’s instructions. The amplification was conducted in a Stratagene Mx3000P machine (MX3000P, Stratagene, USA). The amplification reaction was denaturation at 94 °C for 2 min, followed by 40 cycles of denaturation at 94 °C for 20 s, annealing at 58 °C for 20 s, and extension at 72 °C for 20 s. The relative expression levels were calculated using the 2^−△△Ct^ method [32]. All experiments were repeated three times.

### Statistical Analysis

Data are expressed as mean ± standard deviation. Single comparisons were conducted using an unpaired *t*-test, and *P* < 0.05 was considered statistically significant. GraphPad Prism 8 (GraphPad Software, San Diego, CA, USA) and Image-Pro Plus 6.0 (Media Cybernetics) were used for statistical analysis and visualization.

## Results

### Neurons Ferroptosis Was Activated in Rats Induced by Sepsis

**Fig. 1:**
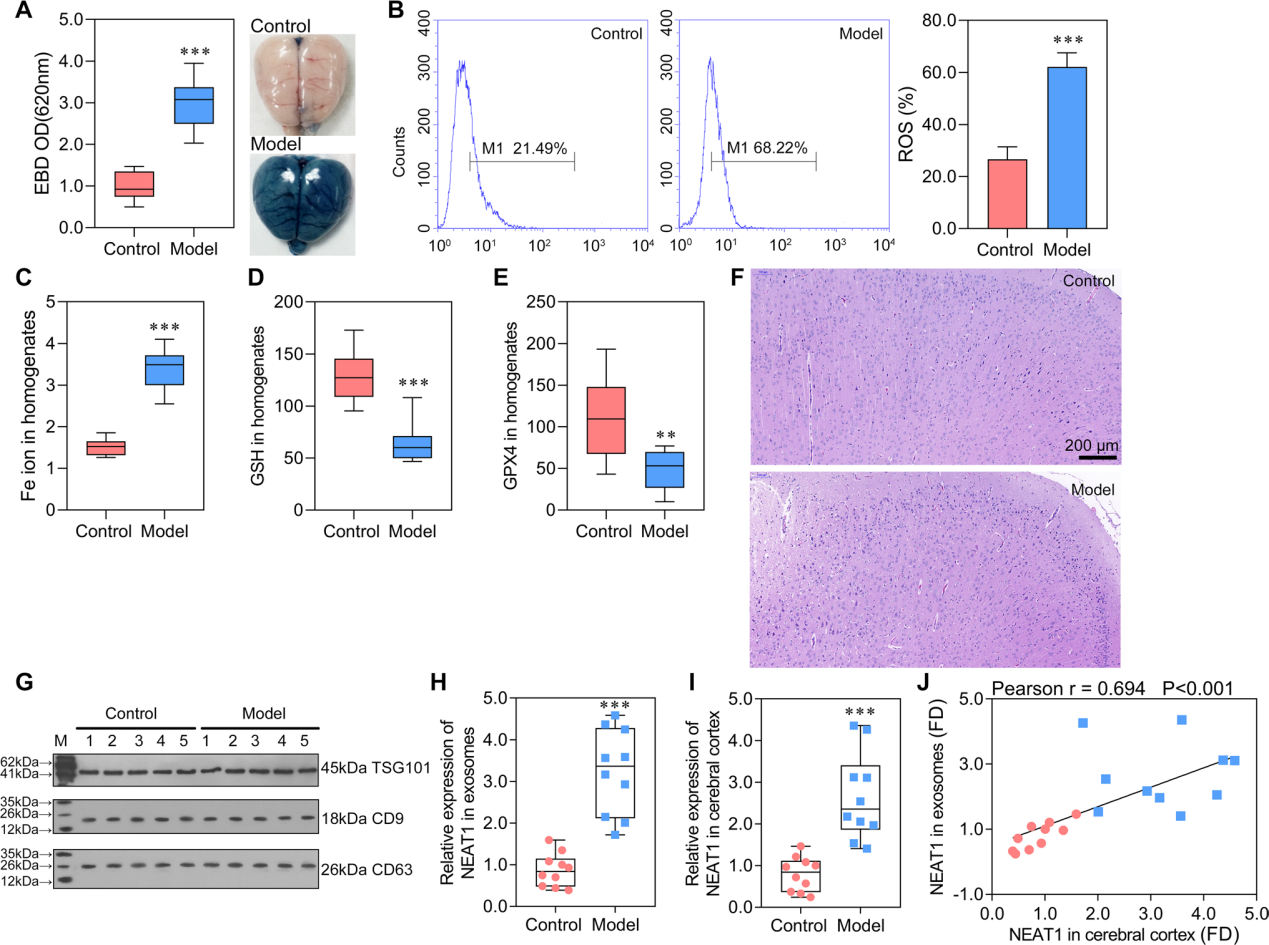
Exosome carried NEAT1 into the cerebral cortex and NEAT1 was significantly highly expressed in sepsis-induced ferroptosis **A** Brain vascular permeability was detected by EBD leakage in control and model rats (*n* = 10). Extracted dye contents in the formamide extracts were quantified at 620 nm; **B** ROS level in cerebral cortex homogenate was detected by flow cytometry. **C**–**E** ELISA analysis on Fe ion, GSH, and GPX4 levels. **F** HE staining on cerebral cortex in control and model rats. **G** Western blot analysis on serous exosome biomarkers of TSG01, CD9, and CD63. **H**, **I** The expression of NEAT1 was detected by qRT-PCR in the serous exosome (normalized to synthetic cel-miR-39-3p) and cerebral cortex (normalized to GAPDH), respectively. **J** The correlation analysis on the expression of NEAT1 in exosome and cerebral cortex. ^**^P < 0.01 vs Control; ^***^P < 0.001 vs Control; EBD, Evans blue dye.

To investigate the occurrence of ferroptosis in sepsis injured brain, the sepsis model was constructed firstly. We can observe from Fig. 1A that the sepsis model was successfully constructed in model rats, in which the EBD was significantly increased, indicating serious BBB damage. Several biomarkers were detected to investigate whether the neurons ferroptosis was induced. ROS and Fe ion levels in cerebral cortex homogenate were significantly increased in model rats compared with control rats (Fig. 1B, C). Additional biomarkers, GSH and GPX4, were significantly decreased in model rats (Fig. 1D, E). The HE staining on observing morphological changes in the neurons in rat models revealed that the number of neurons in model rats were reduced, and the cells were irregularly distributed (Fig. 1F). These results exhibited a successfully constructed ferroptosis model induced by sepsis.

### Exosome-Packaged NEAT1 Was Significantly Upregulated in Ferroptosis Cells

We then purified exosomes from the cerebral cortex and confirmed by identifying the exosome markers of TSG101, CD9, and CD63. They are all expressed in exosomes isolated from control and model rats (Fig. 1G). To further investigate whether exosome package is the leading way to deliver NEAT1, the expression of NEAT1 in exosomes and the cerebral cortex was evaluated. The qRT-PCR revealed that the expression of NEAT1 in exosomes and cerebral cortex were consistent and were significantly higher in the model than in control rats (Fig. 1H, I). Moreover, their expression level of NEAT1 was positively and significantly correlated in the cerebral cortex and exosomes (Fig. 1J). The result hinted that the exosome carrying NEAT1 passed through BBB and reached the cerebral cortex. Combined together, we demonstrated that the expression of NEAT1 was elevated in rats with sepsis-induced ferroptosis. Moreover, exosomes might be the primary way of transporting NEAT1 into the cerebral cortex.

### Sepsis Increases Stress of Ferroptosis in Brain Microvascular Endothelial Cells

**Fig. 2:**
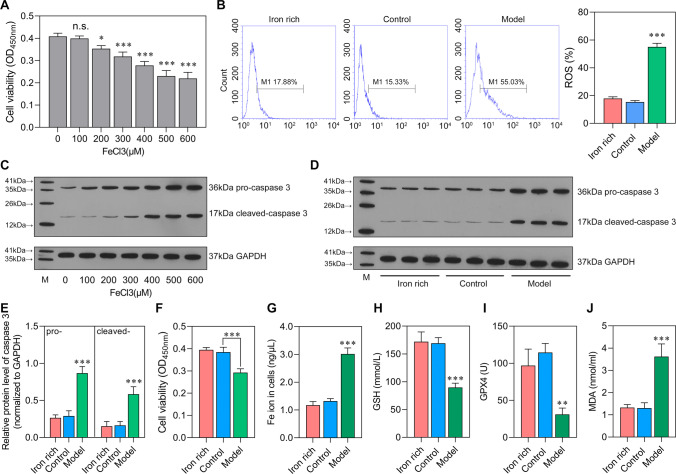
Sepsis increases stress of ferroptosis The bEnd.3 cells were stimulated by FeCl_3_ or serum of rats from control group and sepsis model group. **A** Optimal concentration of FeCl_3_ selection by CCK-8. n.s.: no significance; ^*^P < 0.05, ^**^P < 0.01, ^***^P < 0.001, vs 0 μM FeCl_3_. **B** ROS level in iron-rich (100 μM FeCl_3_), control (100 μM FeCl_3_ + Control serum), and model (100 μM FeCl_3_ + sepsis serum) group was detected by flow cytometry. **C**–**E** The protein levels of pro-caspase 3 and cleaved-caspase 3. **E** Cell viability was detected using CCK-8. **F**–**I** The levels of Fe ion (Fe^2+^ and Fe^3+^), GSH, GPX4, and MDA. *P < 0.05, ^**^P < 0.01, ^***^P < 0.001 vs iron-rich and control.

The cell viability was evaluated on bEnd.3 cultured with graded FeCl_3_. We found that 100 μM FeCl_3_ addition reserved the cell viability which can be chosen as the optimal concentration for inducing in vitro ferroptosis stress model (Fig. 2A). In addition, western blot analysis showed that the protein level of both pro-caspase 3 and cleaved-caspase 3 was increased with the increase of FeCl_3_ concentration (Fig. 2C). Meanwhile, we can observe that an iron-rich group with exosome depleted displayed similar levels of ROS, cell viability, Fe ion, GSH, GPX4, and MDA with the control groups. Of note, the ROS level was higher in the model group than in the iron-rich and control groups (Fig. 2B). The protein levels of pro-caspase 3 and cleaved-caspase 3 were also increased in the model group (Fig. 2D, E). The cell viability was more elevated in the iron-rich and control groups than in the model group (Fig. 2F). Fe ion and MDA levels were significantly increased in the model group than in the iron-rich and control groups (Fig. 2G, J). Compared with the iron-rich and control groups, the levels of GSH and GPX4 were significantly increased in that of model (Fig. 2H, I). In summary, the in vitro analysis confirmed the role of sepsis-related factors on transporting Fe ion into brain microvascular endothelial cells.

### NEAT1 Functions as a ceRNA for miR-9-5p to Facilitate TFRC and GOT1 Expression

**Fig. 3:**
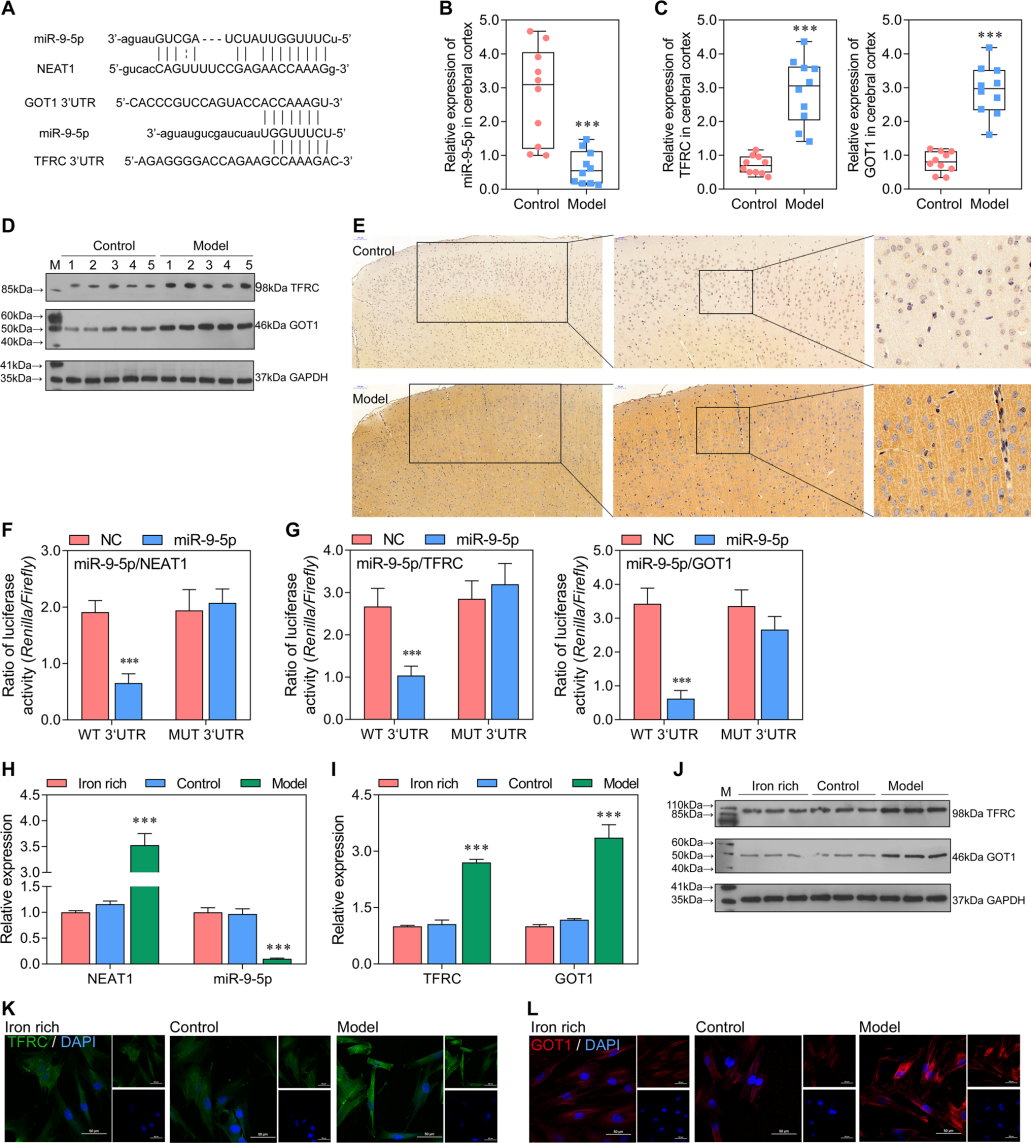
miR-9-5p, sponged by NEAT1, overexpressed in vitro and in vivo and might promote cell apoptosis in sepsis-induced ferroptosis by targeting *TFRC* and *GOT1 A* The binding regions between miR-9-5p with sequences from NEAT1, *TFRC*, and *GOT1*. **B**, **C** The expression level of miR-9-5p, *TFRC*, and *GOT1* in model and control was tested by qRT-PCR in vivo (normalized to *U6* or *GAPDH*). ^***^P < 0.001 vs control. **D** The protein levels of TFRC and GOT1 in cerebral cortex were assessed using Western blotting. **E** The protein level of TFRC in cerebral cortex was showed by immunohistochemistry. **F**, **G** The dual-luciferase reporter gene assay analyzes the binding relationship between miR-9-5p with sequences from NEAT1, *TFRC*, and *GOT1*. ^***^P < 0.001 vs NC. **H**, **I** The expression level of miR-9-5p, *TFRC*, and *GOT1* in iron-rich (100 μM FeCl_3_), control (100 μM FeCl_3_ + control serum), and model (100 μM FeCl_3_ + sepsis serum) group was tested by qRT-PCR (normalized to *U6* or *GAPDH*). ^*^P < 0.05, ^**^P < 0.01, ^***^P < 0.001 vs iron-rich and control. **J** The protein levels of TFRC and GOT1 assessed using western blotting in vitro. **K**, **L** The immunofluorescence for detecting TFRC and GOT1 (blue, DAPI; green, TFRC; red, GOT1).

To explore whether exosome derived NEAT1 functions in sepsis-induced ferroptosis, we investigated potential downstream signaling molecule of NEAT1 in bEnd.3 cells. We can observe from Fig. 3A that miR-9-5p has binding regions with sequences from NEAT1, *TFRC*, and *GOT1*. Moreover, the expression of miR-9-5p was significantly inhibited, while that of *TFRC* and *GOT1* was significantly increased in the model group (Fig. 3B, C). The levels of TFRC and GOT1 were also increased in model rats assessed by western blot (Fig. 3D). Increased TFRC level was individually displayed by immunohistochemistry (Fig. 3E). Moreover, the dual-luciferase reporter gene assay verified the sponging role of NEAT1 on miR-9-5p (Fig. 3F). *TFRC* and *GOT1* were also confirmed as the target genes of miR-9-5p through dual-luciferase reporter gene assay (Fig. 3G). The expression levels of miR-9-5p, NEAT1, *TFRC*, and *GOT1* were consistent between the iron-rich and control group in bEnd.3 cells (Fig. 3H, I). Besides, their expression levels detected in vitro were also consistent with that in vivo, in which miR-9-5p have opposed expression trend with NEAT1, *TFRC*, and *GOT1* in model compared with iron-rich and control. Agreed with gene expression, the protein levels of TFRC and GOT1 were higher in model than iron-rich and control (Fig. 3J–L). Taken together, the result revealed that miR-9-5p, sponged by NEAT1, targeted *TFRC* and *GOT1* that overexpressed in vitro and in vivo and might promote sepsis-induced ferroptosis in brain microvascular endothelial cells.

### Increased miR-9-5p Regulates the Expression of TFRC and GOT1 and Relieves Ferroptosis

**Fig. 4:**
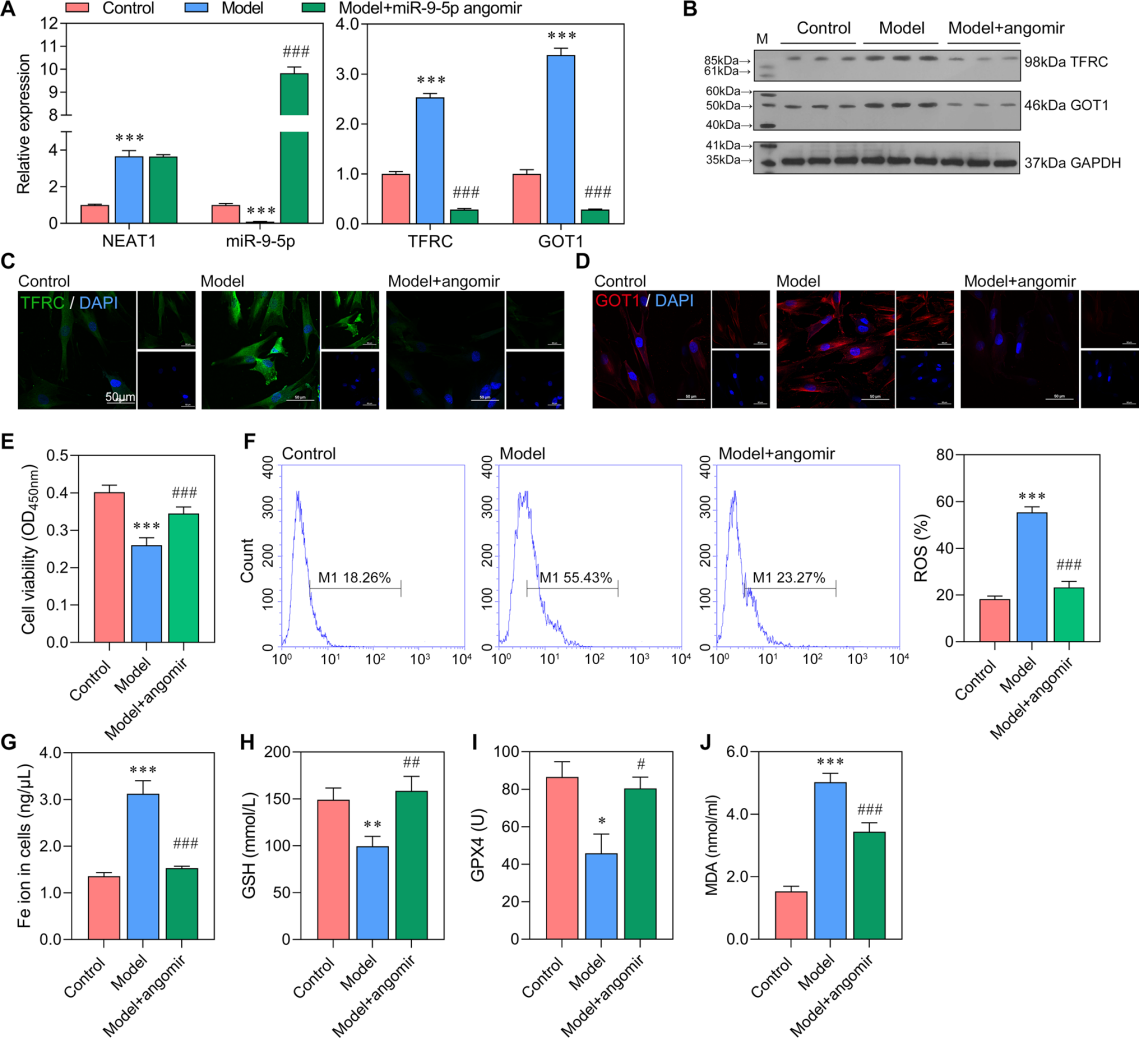
Increased miR-9-5p regulates the expression of *TFRC* and *GOT1* and relieves ferroptosis in vitro The bEnd.3 cells were transfected with miR-9-5p angomir, followed by serum stimulation. **A** The expression levels of NEAT1, miR-9-5p, *TFRC*, and *GOT1* in control, model, and model + miR-9-5p angomir were tested by qRT-PCR (normalized to *U6* or *GAPDH*). **B** The sequence for NEAT1 mutant. **C** The protein levels of TFRC and GOT1. **D**, **E** The immunofluorescence for TFRC and GOT1 (blue, DAPI; green, TFRC; red, GOT1). **F** The cell viability of bEnd.3 cells were evaluated by CCK-8 assay. **G**–**K** The levels of ROS, Fe ion (Fe^2+^ and Fe^3+^), GSH, GPX4, and MDA were assessed. ^*^P < 0.05, ^**^P < 0.01, ^***^P < 0.001 vs control; ^#^*P* < 0.05, ^##^*P* < 0.01, ^###^*P* < 0.001 vs model.

The ceRNA network among NEAT1, miR-9-5p, *TFRC*, and *GOT1* was evaluated by adding miR-9-5p angomir in bEnd.3cells treated with exosome isolated from serum of control and model rats. The miR-9-5p angomir significantly increased the expression of miR-9-5p and have no significant effect on that of NEAT1 (Fig. 4A). With the addition of miR-9-5p, the expression of *TFRC* and *GOT1* were significantly decreased, which was negatively regulated by miR-9-5p. Besides, the protein expression tendency of TFRC and GOT1 in control, model, and model + miR-9-5p angomir were consistent with that of mRNA expression tendency (Fig. 4B–D). These results revealed that increased miR-9-5p, targeting TFRC and GOT1, negatively regulates their expression levels. Furthermore, compared with model cells, those supplemented with miR-9-5p angomir have higher cell viability (Fig. 4E). Then, we assessed the markers of ferroptosis and found that the levels of ROS, Fe ion, GSH, GPX4, and MDA in the model + miR-9-5p angomir group came close to that in the control group and significantly different from that in the model group (Fig. 4F–J). ROS, Fe ion, and MDA levels were significantly reduced in cells supplemented with miR-9-5p angomir. The levels of GSH and GPX4 were significantly higher in the model + miR-9-5p angomir group than in the model group. Combined together, we can conclude that increased miR-9-5p relieved ferroptosis and regulated the expression levels of *TFRC* and *GOT1*.

### Enhanced NEAT1 Promoted Ferroptosis and Increased the Expression of TFRC and GOT1

**Fig. 5:**
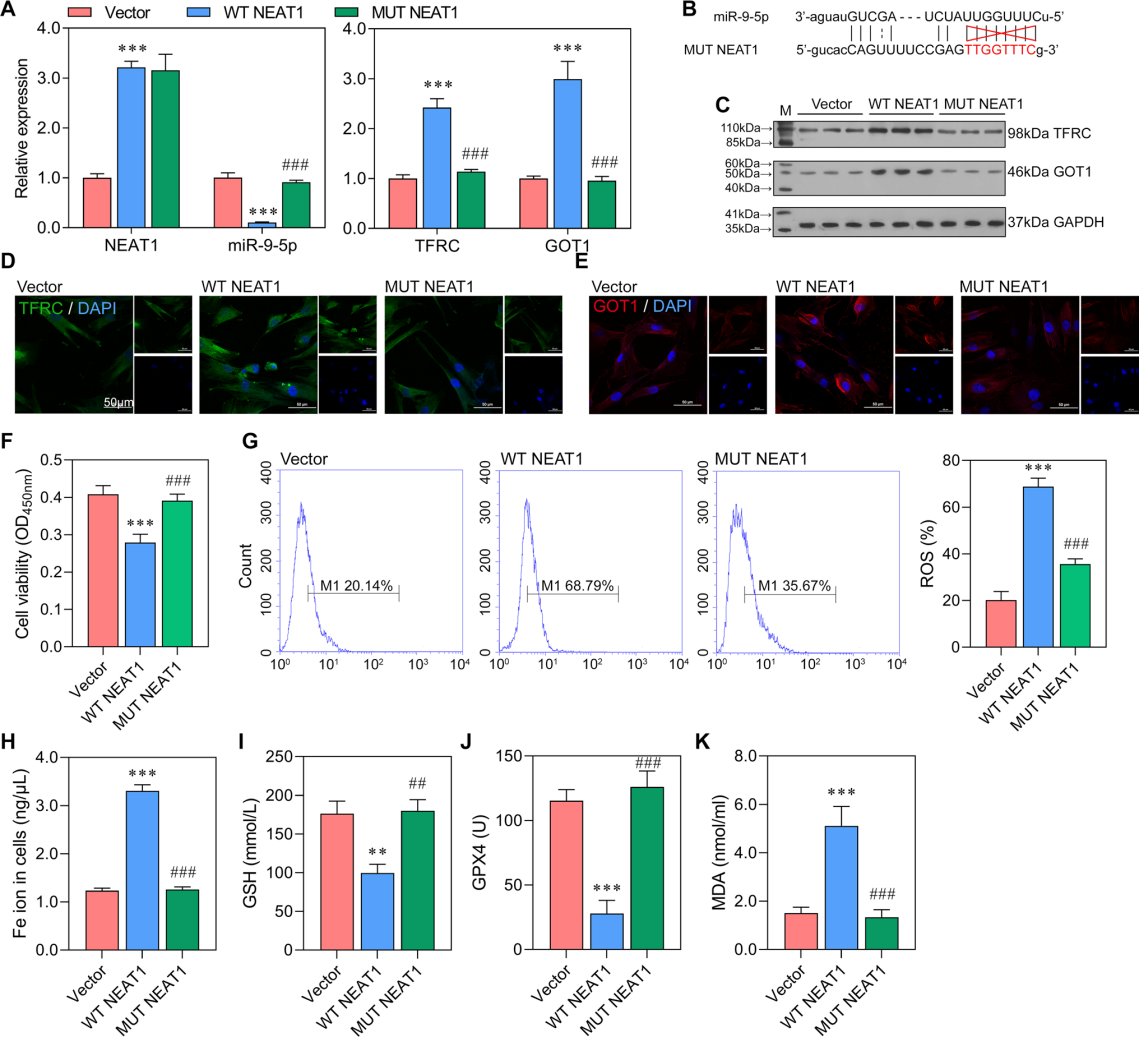
Enhanced NEAT1 promoted ferroptosis and increased the expression of *TFRC* and *GOT1* in vitro NEAT1 was overexpressed in bEnd.3 cells. **A** The expression levels of NEAT1, miR-9-5p, *TFRC*, and *GOT1* in Vector, WT NEAT1 (wild NEAT1 sequence), and MUT NEAT1 (site-directed mutant NEAT1) by qRT-PCR (normalized to *U6* or *GAPDH*). **B** The protein levels of *TFRC* and *GOT1* showed by western blotting. **C**, **D** The immunofluorescence for TFRC and GOT1 (blue, DAPI; green, TFRC; red, GOT1). **E** The cell viability of cells detected by CCK-8 assay. **F**–**J** The levels of ROS, Fe ion (Fe^2+^ and Fe^3+^), GSH, GPX4, and MDA. ^**^P < 0.01, ^**^P < 0.01, ^***^P < 0.001 vs Vector; ^##^*P* < 0.01, ^###^*P* < 0.001 vs WT NEAT1.

To further investigate NEAT1 role in ferroptosis, WT NEAT1 and MUT NEAT1 were overexpressed in bEnd.3cells. The expression level of NEAT1 in WT NEAT1 group was close to that in MUT NEAT1 group, which were higher than that in Vector group (Fig. 5A). The WT NEAT1 negatively regulated the expression of miR-9-5p and positively regulated the expression of *TFRC* and *GOT1* (*P* < 0.001; Fig. 5A). The western blot analysis verified the result of qRT-PCR, in which the protein levels of TFRC and GOT1 were higher in WT NEAT1 than those in Vector and MUT NEAT1 groups (Fig. 5C). The immunofluorescence also proved that *TFRC* and *GOT1* were highly expressed in WT NEAT1 compared with Vector and MUT NEAT1 groups (Fig. 5D, E). The result further confirmed the ceRNA network of NEAT1/miR-9-5p/*TFRC* and *GOT1*. In addition, the cell viability was lower in MUT NEAT1 than in Vector and MUT NEAT1 groups (Fig. 5F). The levels of ROS, Fe ion, GSH, GPX4, and MDA in WT NEAT1 are significantly different from Vector and MUT NEAT1 groups (Fig. 5G–K), which indicate that increased NEAT1 promoted ferroptosis. Combined together, it suggested that elevated NEAT1 increased the expression of *TFRC* and *GOT1* by sponging miR-9-5p, thus inducing ferroptosis.

### *miR-9-5p Alleviated Sepsis-Induced Ferroptosis by Suppressing the Expression of TFRC and GOT1 *In Vivo

**Fig. 6:**
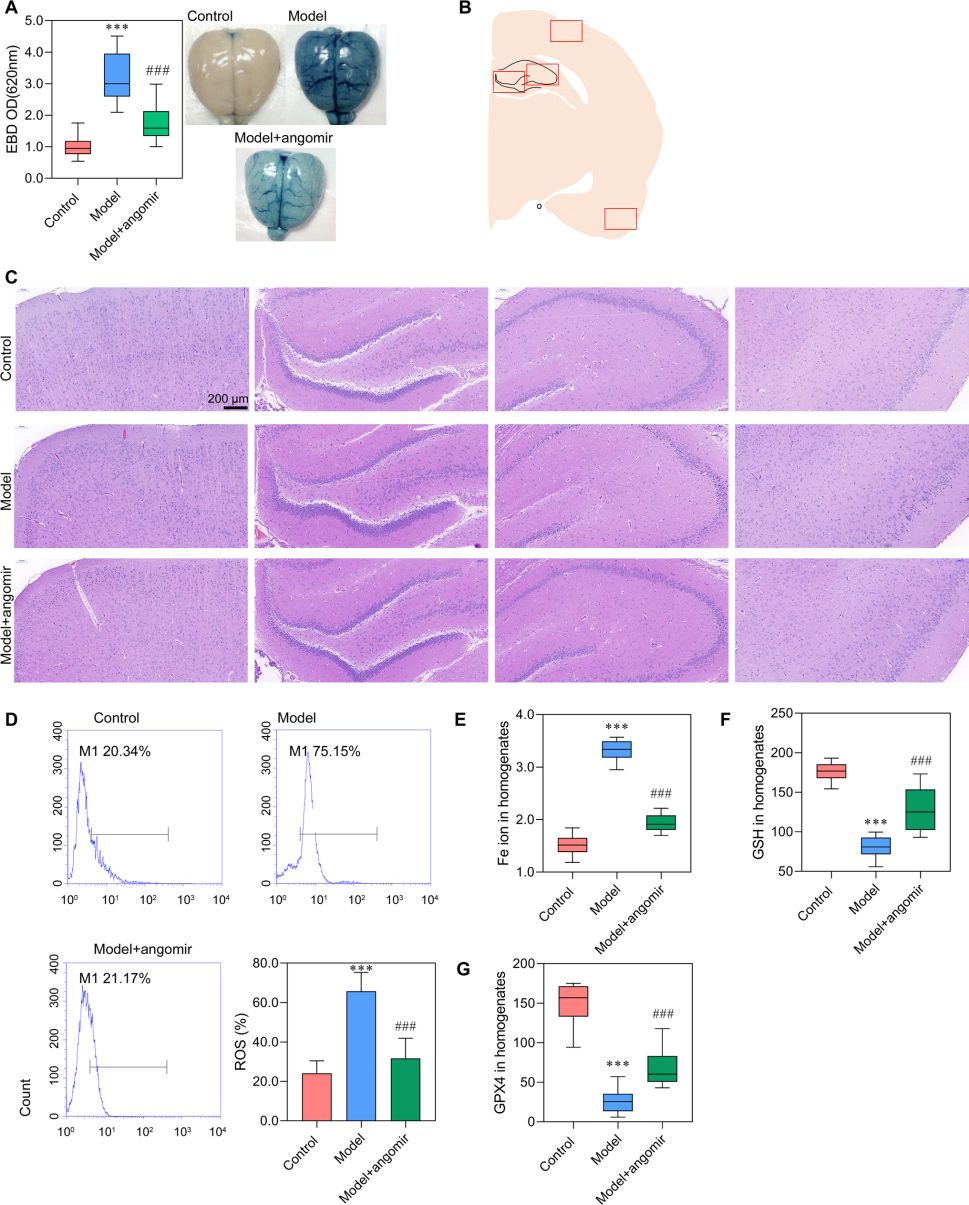
miR-9-5p alleviated ferroptosis in vivo Sepsis model rats were administered with miR-9-5p angomir through tail vein injection. **A** Brain vascular permeability was detected by EBD leakage in control, model, and model + miR-9-5p angomir rats (*n* = 10). Extracted dye contents in the formamide extracts were quantified at 620 nm. **B**, **C** HE staining of four brain regions. **D** ROS level in cerebral cortex homogenate was detected by flow cytometry. **E**–**G** ELISA analysis on Fe ion, GSH, and GPX4 levels. ^***^P < 0.001 vs control; ^###^*P* < 0.001 vs model.

**Fig. 7:**
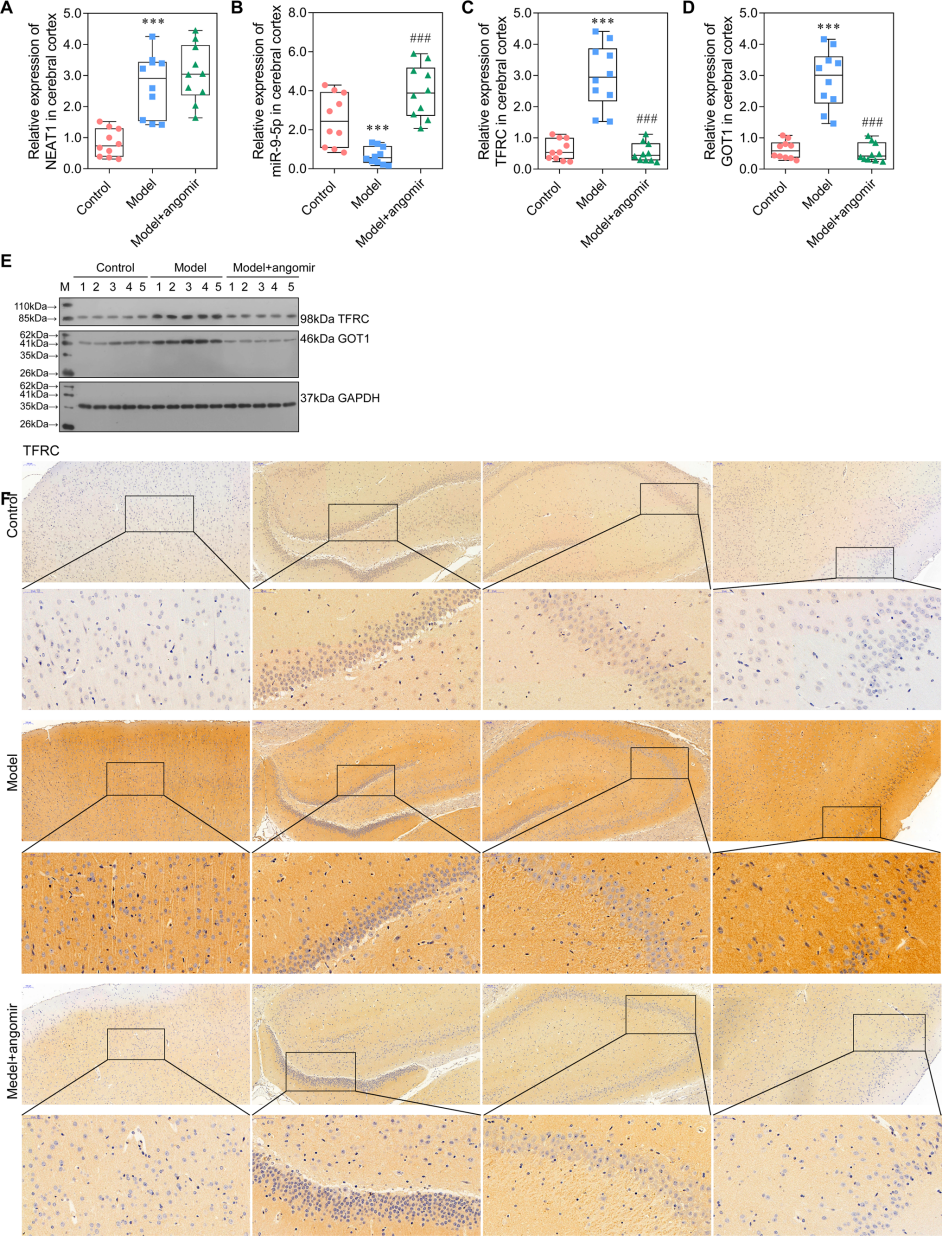
miR-9-5p suppressed the expression of *TFRC* and *GOT1* in vivo **A**–**D** The qRT-PCR analysis on the expression of NEAT1, miR-9-5p, *TFRC*, and *GOT1* in the cerebral cortex of control, model, and model + miR-9-5p angomir rats, respectively (normalized to *U6* or *GAPDH*). **E** The western blotting analysis on TFRC and GOT1. **F** The protein level of TFRC in four brain regions were assessed by Immunohistochemistry. ^***^P < 0.001 vs control; ^###^*P* < 0.001 vs model.

**Fig. 8:**
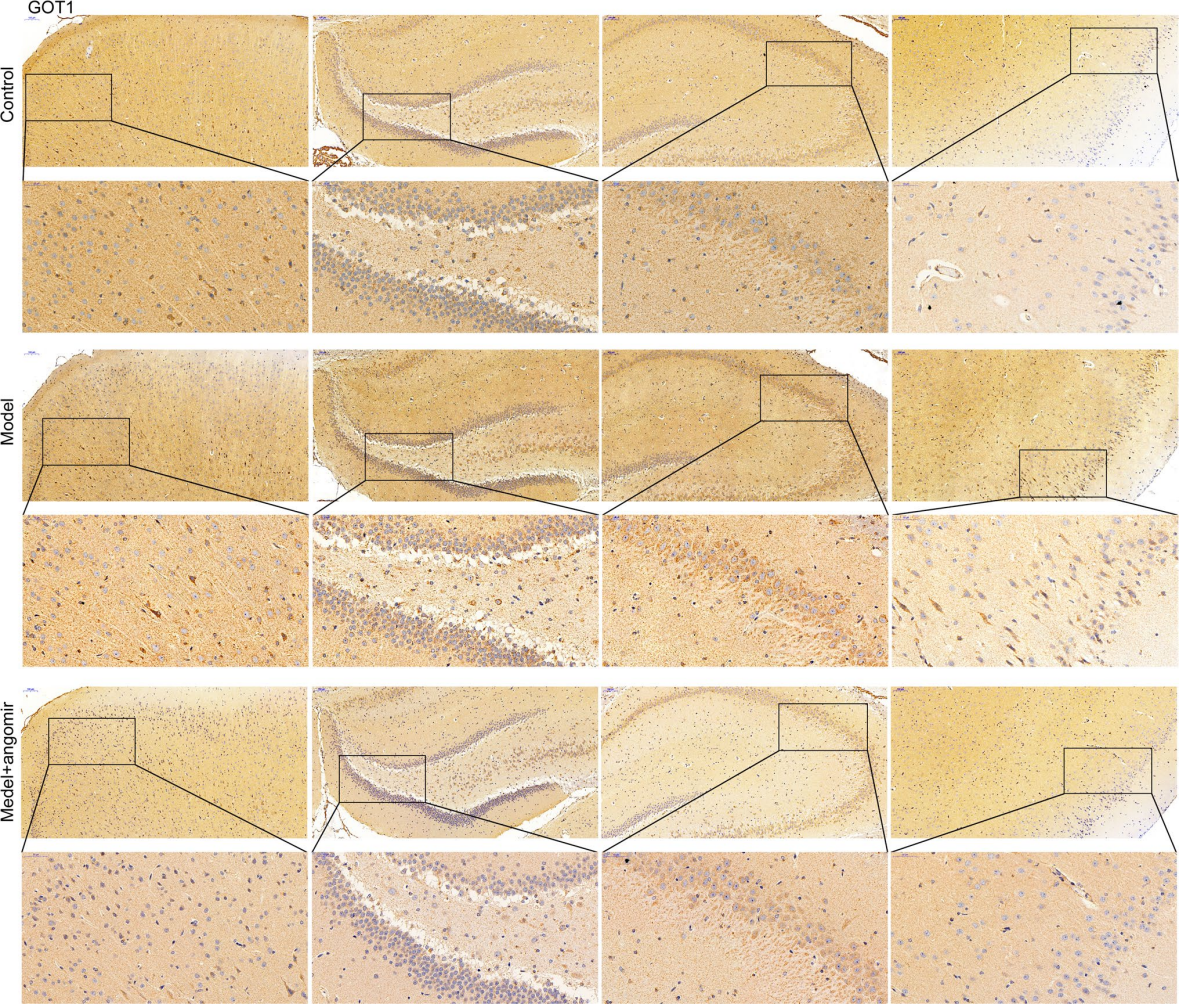
miR-9-5p suppressed the expression of and *GOT1* in vivo The protein level of GOT1 in four brain regions was assessed by Immunohistochemistry.

In vivo experiment was performed to confirm the result obtained in in vitro study. As observed in Fig. 6A, miR-9-5p angomir significantly reduced the EBD in the model + miR-9-5p angomir group compared with the model group (Fig. 6A). Furthermore, we collected the four regions of brain tissue and performed HE staining (Fig. 6B, C). The neurons increased in model + miR-9-5p angomir rats, and the arrangement of cells is more orderly, when compared with the model rats. The levels of ROS, Fe ion, GSH, GPX4, and MDA at model + miR-9-5p angomir rats came close to that of control rats and significantly differed from that in model rats (Fig. 6D–G), which indicated that increased miR-9-5p in rats alleviated sepsis-induced ferroptosis. Moreover, the expression level of NEAT1 was no change with supplementary miR-9-5p angomir when compared with that in the model group (Fig. 7A), which was consistent with that in vitro. The expression of miR-9-5p was significantly increased in the model + miR-9-5p angomir rats compared with the model rats (Fig. 7B). The RT-PCR, western blot, and immunohistochemistry analyses demonstrated that the expression levels of *TFRC* and *GOT1* were significantly decreased with the addition of miR-9-5p angomir (Fig. 7C–F, Fig. 8). Taken together, the in vivo analysis verified that increased miR-9-5p, sponged by NEAT1, alleviated ferroptosis by suppressing the expression of *TFRC* and *GOT1*.

## Discussion

Ferroptosis is a severe cell death program, which can cause several diseases, including SAE. We investigate the ceRNA axis NEAT1/miR-9-5p/*TFRC*/*GOT1* on sepsis-induced ferroptosis for the first time in the present study. The present study drew the following conclusions: (1) neurons ferroptosis can be activated by sepsis in rats; (2) NEAT1, packaged in exosome, passed through BBB and highly expressed in the cerebral cortex of rats with ferroptosis; (3) NEAT1 functions as a ceRNA for miR-9-5p to facilitate *TFRC* and *GOT1* expression; (4) miR-9-5p alleviated sepsis-induced neurons ferroptosis by suppressing the expression of *TFRC* and *GOT1*; and (5) the lncRNA NEAT1 might regulates SAE through miR-9-5p/*TFRC*/*GOT1*.

Our present study verified the exosome role in transporting NEAT1 into the cerebral cortex through BBB, consistent with that reported by other researchers fz. Morales-Prieto et al. [33] indicated that exosomes could pass through BBB and act on glial cell activation. Reynolds et al. [34] revealed that human microglial cells exosomes cross the BBB and are efficient delivery vehicles to the CNS. More importantly, we demonstrated that exosome-delivering is the main way for transporting lncRNA NEAT1 into the cerebral cortex. The stability and transporting features of the exosome provide a solid foundation for subsequent experiments.

Liu et al. [35] also demonstrated that NEAT1 promotes brain injury in septic mice by positively regulating the NF-κB pathway. Moreover, they showed that NEAT1 could be an essential diagnostic marker and therapeutic target for brain injury induced by sepsis. However, ferroptosis has not been studied. Our present study further explored the relationship among brain injury, sepsis, and ferroptosis. We found that exosomal NEAT1 transported into the cerebral cortex and functions as a ceRNA for miR-9-5p to facilitate *TFRC* and *GOT1* expression, therefore, play important roles in sepsis-induced ferroptosis and SAE. Our present study was revealed the relationship between SAE and ferroptosis for the first time. Not only brain injury, NEAT1 also plays an important role in sepsis-induced liver injury by promoting inflammatory responses via the ceRNA regulatory axis [36]. In some other studies, NEAT1 was reported to be a valuable biomarker functions in various diseases by sponging miRNAs. For example, NEAT1, sponging miR-9-5p, enhances the resistance of anaplastic thyroid carcinoma cells to cisplatin by regulating SPAG9 expression [37], promotes the growth of cervical cancer cells [16], and regulates pulmonary fibrosis [38]. NEAT1 suppresses ferroptosis by competing with miR-34a-5p and miR-204-5p and promoting the transcription of *ACSL4* [39]. The functions of NEAT1 varied in different axis and diseases. We suspected that NEAT1 might be a valuable biomarker for the identification of sepsis-induced ferroptosis and SAE.

As the sponging miRNA of NEAT1, miR-9 was initially identified as a brain-specific miRNA and implicated in mammalian diseases concerns about neuronal development and function, including Alzheimer’s and Huntington’s [40]. miR-9-related regulatory axis engaged in the inflammatory response and inflammation-related diseases [41, 42] and involved in sepsis-associated acute kidney injury [43, 44]. Moreover, Sun et al. [45] demonstrated that ferroptosis play a positive role in inflammation through immunogenicity. Therefore, we assumed that miR-9 might regulate SAE through regulating inflammation, which will be studied in our future work. In the present study, we found that miR-9-5p alleviated sepsis-induced neurons ferroptosis by suppressing the expression of *TFRC* and *GOT1*.

To investigate the role of NEAT1, *TFRC*, *GOT1*, and miR-9-5p on sepsis-induced ferroptosis and SAE, some of the biomarkers, as ROS, GPX4, GSH, and MDA, were tested. Ferroptosis is characterized by inflammation, detrimental lipid ROS formation, disrupted GPX4 redox defense, and GSH depletion [46, 47]. The production of ROS is closely related to the disturbance of iron homeostasis. The ferroptosis can be blocked by iron-chelating agents, indicating a tight relationship between intracellular iron and ferroptosis [48]. These toxic lipid peroxides can be converted into non-toxic alcohols under the action of GSH and GPX4, thereby avoiding their killing effects on cells [49]. Ferroptosis is specifically activated by missing GPX4 activities [50]. In the present study, the levels of GPX4 were significantly decreased in sepsis-induced ferroptosis rats. MDA accumulation can cause the cross-linking and polymerization of proteins and nucleic acids and destroy the membrane structure, thus leading to cell death [51]. In the present study, ferroptosis induced ROS production, GSH depletion, and disrupted GPX4 function, consistent with that reported in other studies [46, 52, 53].

In the beginning, ferroptosis was thought to be a cell death pathway different from apoptosis, necrosis, and autophagy at the biochemical, morphological, and genetic levels [54]. However, more and more evidence showed that the occurrence of ferroptosis requires the participation of autophagy mechanisms [55]. Hou et al. [56] revealed the relationship between autophagy and ferroptosis for the first time by knocking down ATG5 and ATG7 in vivo, in which the intracellular free iron and MDA levels were significantly decreased, and *FTH1* was significantly increased. Although the free iron and MDA levels were significantly decreased in sepsis-induced ferroptosis in the present study, the expression level of *FTH1* was lacked to be tested, which needed to be evaluated in our future study. Zhou et al. [57] confirmed the relationship between autophagy and ferroptosis and further concluded that autophagy process degrades ferritin and increases free iron in cells, thus promoting ferroptosis. Since then, many studies have confirmed that macromolecular substances as GSH, GPX4, and lipid peroxides are closely related to ferroptosis and involved in the occurrence of autophagy [58, 59]. Studies have shown that the occurrence of autophagy is accompanied by a decrease in GSH under starvation or oxidation conditions [58]. Moreover, overexpression of GPX can inhibit the occurrence of ROS-mediated autophagy [59]. Although more and more studies have confirmed that autophagy can promote ferroptosis, some other studies demonstrated that ferroptosis can be independent of autophagy.

Kremer et al. [60] demonstrated that *GOT1* withdrawal was reported to promote a catabolic cell state, resulting in decreased OxPHOS, activated autophagy, and ferritinophagy, which raised iron pools and promoted ferroptosis. Therefore, we assumed that autophagy is related to sepsis-induced ferroptosis. The increase of iron ions in the brain induces the iron death of glial cells or hippocampus cells and finally induces SAE. As reported, miR-9 regulates ferroptosis by targeting *GOT1* in melanoma [22]. Overexpressed miR-9 suppressed *GOT1* by binding to its 3′-UTR, which subsequently reduced ferroptosis. In the present study, we concluded that miR-9-5p regulates sepsis-induced ferroptosis by targeting *GOT1*, thus causing SAE. As reported, *TFRC* can significantly increase the intracellular iron load, and its upregulation confers cell sensitivity to ferroptosis induced by GPX4 inhibition [61]. *TFRC* is a crucial mediator for ferroptosis [62]. Through *TFRC*-mediated endocytosis, the iron carrier protein transferrin can be transported into cells. Silencing *TFRC* can inhibit conditional serum-induced ferroptosis significantly [63]. Therefore, *TFRC* might participate in sepsis-induced ferroptosis by increasing the iron content in cells. The whole study demonstrated that exosome-mediated lncRNA NEAT1 passes through the BBB and exacerbates SAE by promoting ferroptosis through NEAT1/miR-9-5p/*TFRC* and *GOT1* axis.

## Conclusion

The present study demonstrated that NEAT1 in exosomes sponged miR-9-5p, which promoted the expression of *TFRC* and *GOT1*. The overexpressed *TFRC* and *GOT1* could induce ferroptosis in the brain microvascular endothelial cells and finally induce SAE. The present study provided a clue for SAE of sepsis-induced ferroptosis through axis NEAT1/miR-9-5p/*TFRC* and *GOT1*.

## Supplementary Information

Below is the link to the electronic supplementary material.

Supplementary file1 (TIF 5195 KB)

Supplementary file2 (DOCX 16 KB)

## Data Availability

All data generated or analyzed in this study are available in the published article.
