# Correction to: Exosome-Derived lncRNA NEAT1 Exacerbates Sepsis-Associated Encephalopathy by Promoting Ferroptosis Through Regulating miR-9-5p/ *TFRC* and *GOT1* Axis

**DOI:** 10.1007/s12035-022-02931-2

**Published:** 2022-06-21

**Authors:** Xue-biao Wei, Wen-qiang Jiang, Ju-hao Zeng, Lin-qiang Huang, Hong-guang Ding, Yuan-wen Jing, Yong-li Han, Yi-chen Li, Sheng-long Chen

**Affiliations:** 1 Department of Geriatric Intensive Care Unit, Guangdong Provincial Geriatrics Institute, Guangdong Provincial People’s Hospital, Guangdong Academy of Medical Sciences, Guangzhou, People’s Republic of China; 2 Department of Critical Care Medicine, Guangdong Provincial People’s Hospital, Guangdong Academy of Medical Sciences, 106 Zhongshan Er Road, Guangzhou, 510080 People’s Republic of China

**Correction to: Mol Neurobiol (2022) 59(3):1954-1969**

10.1007/s12035-022-02738-1

The original version of this article unfortunately contained a writing mistake in the first paragraph of Material and Methods. The “C57BL/6 rats” should be “Sprague Dawley rats”.

