# Supplementary figures and images for: Exosome-Derived lncRNA NEAT1 Exacerbates Sepsis-Associated Encephalopathy by Promoting Ferroptosis Through Regulating miR-9-5p/TFRC and GOT1 Axis
Source: Mol Neurobiol. 2022 Jan 17;59(3):1954–69. doi: 10.1007/s12035-022-02738-1 (PMC8882117; doi:10.1007/s12035-022-02738-1)

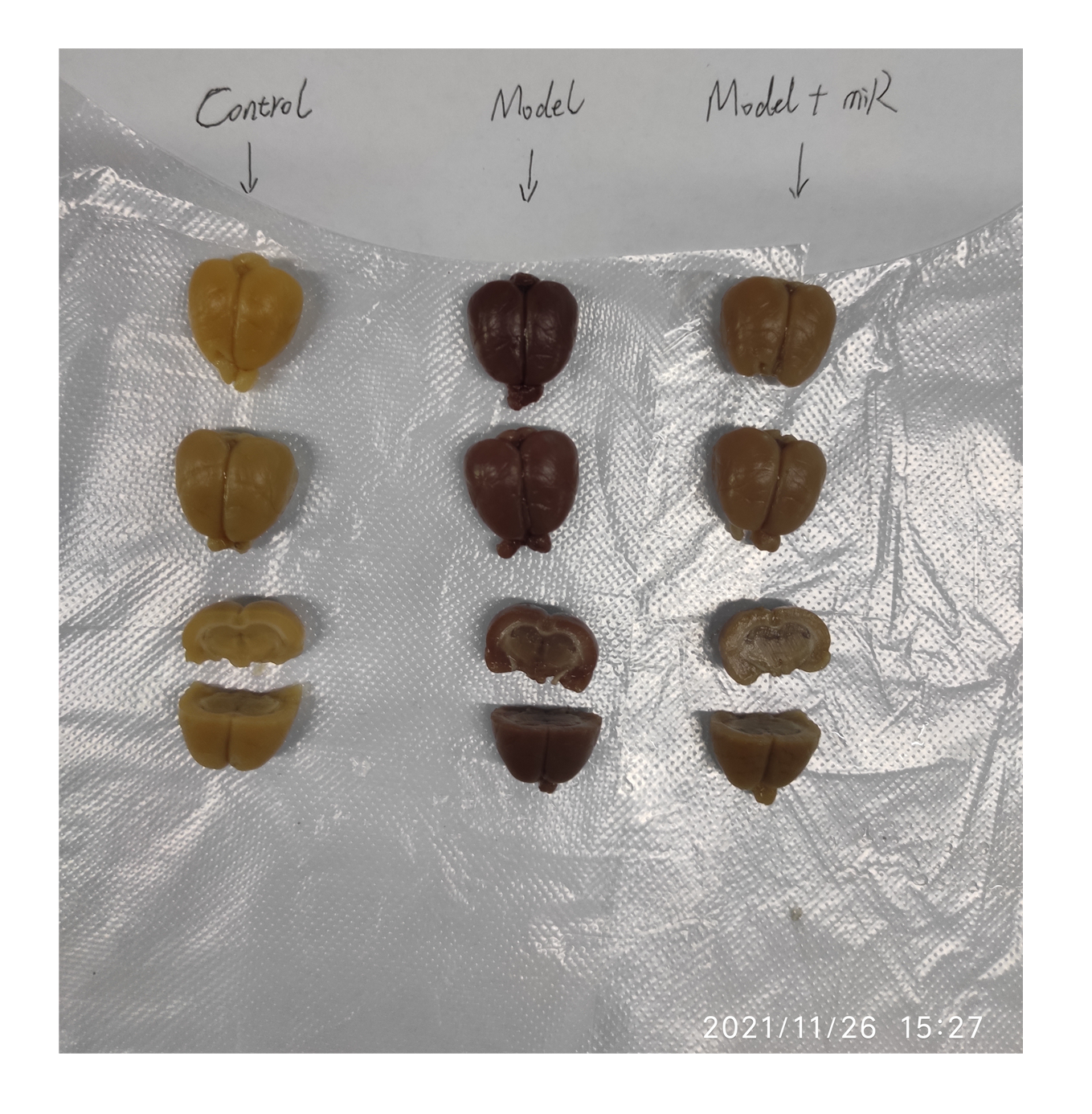

Supplement: Supplementary file 1 — Supplementary file1 (TIF 5195 KB) [file 12035_2022_2738_MOESM1_ESM.tif]
