# Supplementary material for: Exosome-Derived lncRNA NEAT1 Exacerbates Sepsis-Associated Encephalopathy by Promoting Ferroptosis Through Regulating miR-9-5p/TFRC and GOT1 Axis
Source: Mol Neurobiol. 2022 Jan 17;59(3):1954–69. doi: 10.1007/s12035-022-02738-1 (PMC8882117; doi:10.1007/s12035-022-02738-1)
Supplement: Supplementary file 2 — Supplementary file2 (DOCX 16 KB) [file 12035_2022_2738_MOESM2_ESM.docx]

Table S1. The primer sequences list for miRNA, lncRNA, or mRNAs.

| Name | Primer sequence |
| --- | --- |
| miR-9-5p RT | CTCAACTGGTGTCGTGGAGTCGGCAATTCAGTTGAGTCATACAG |
| miR-9-5p F | ACACTCCAGCTGGGTCTTTGGTTATCTAGCTG |
| miR-9-5p R | CTCAACTGGTGTCGTGGA |
| NEAT1 F | GTCAGACACTGGATGGTGGG |
| NEAT1 R | GCAAGACAAGGTGTGGCAAG |
| TFRC F | TGGTTCGTACAGCAGCAGAG |
| TFRC R | GCACGGAAGTAGTCTCCACG |
| GOT1 F | GAAGACAATGGCTGACCGGA |
| GOT1 R | AGGTTCTTGGTGGTCAAGCC |
| GAPDH F | CCTCGTCTCATAGACAAGATGGT |
| GAPDH R | GGGTAGAGTCATACTGGAACATG |
| U6 F | CTCGCTTCGGCAGCACAT |
| U6 R | AACGCTTCACGAATTTGCGT |
| cel-miR-39-3p RT | CTCAACTGGTGTCGTGGAGTCGGCAATTCAGTTGAGCAAGCTGA |
| cel-miR-39-3p F | ACACTCCAGCTGGGTCACCGGGTGTAAATCAG |
| cel-miR-39-3p R | CTCAACTGGTGTCGTGGA |

RT: reverse transcription; F: foword primer; R: reverse primer; GAPDH, U6 and cel-miR-39-3p was used as reference genes.
